# The Research of New Natural Spontaneous Fertile *Attention: Title Altered* Hybrids (*Aegilops trivialis* Migusch. Et Chak) Using Laser Microscopy and Tandem Mass Spectrometry

**DOI:** 10.3390/ijms27114758

**Published:** 2026-05-25

**Authors:** Nadezhda N. Chikida, Mayya P. Razgonova, Muhammad Amjad Nawaz, Maria Kh. Belousova, Kirill S. Golokhvast

**Affiliations:** 1 N.I. Vavilov All-Russian Institute of Plant Genetic Resources, B. Morskaya 42-44, Saint-Petersburg 190000, Russia; n.chikida@mail.ru (N.N.C.); m.h.belousova@mail.ru (M.K.B.); 2 Advanced Engineering School, Institute of Biotechnology, Bioengineering and Food Systems, Far Eastern Federal University, Fr. Russian, pos. Ajax, 10, Vladivostok 690922, Russia; 3 Higher Engineering School of Agrobiotechnology, National Research Tomsk State University, Lenin Ave, 36, Tomsk 634050, Russia; golokhvast@sfsca.ru; 4 Laboratory for Research and Application of Supercritical Fluid Technologies in Agro-Food Biotechnology, National Research Tomsk State University, Lenin Ave, 36, Tomsk 634050, Russia; 5 Siberian Federal Scientific Centre of Agrobiotechnology, Centralnaya, Presidium, Krasnoobsk 633501, Russia

**Keywords:** ear, spikelets, grain, electrophoretic analysis, gliadin spectrum, protein formula, laser microscopy, tandem mass spectrometry, spontaneous hybrids, *Aegilops trivialis* Migusch. Et Chak

## Abstract

The study of natural spontaneous fertile hybrids, whose parent species is *Ae. trivialis* Migusch. et Chak (2n = 42), is of great importance for expanding the genetic pool of the genus *Triticum* L., which is a crucial part of current and future breeding efforts. The number of wild relatives—potential sources of valuable disease resistance genes—is quite large for common wheat: these include species of the genera *Tritium*, *Aegilops*, *Agropiron*, *Secale*, *Haynaldia*, *Villosa*, and others. In addition to disease and pest resistance, wild species offer frost resistance, drought tolerance, salt tolerance, and increased protein quantity and quality. The primary objective of this study was to identify new, genetically diverse source material for common wheat breeding based on botanical and morphological studies, as well as to register new spontaneous Aegilops–wheat hybrids using electrophoretic analysis of storage proteins. To achieve the research objective, the following tasks were set and solved: Aegilops–wheat hybrids were studied and recorded using protein formulas; spontaneous fertile Aegilops–wheat hybrids were analyzed using laser microscopy and tandem mass spectrometry. In this study, we demonstrated differences between the studied spontaneous hybrids using metabolomic analysis and laser microscopy, as well as identified differences in the protein spectra of the spontaneous hybrids and their maternal form, K-1386. These spontaneous Aegilops–wheat hybrids will be used in further work to identify their paternal form. It should be noted that it is advisable to use the studied spontaneous Aegilops–wheat hybrids in future breeding to expand the gene pool of the genus *Triticum* L. and to obtain new heterogeneous forms.

## 1. Introduction

There are only a limited number of wheat relatives whose chromosomes have the ability to pair with the chromosomes of the wheat genome, which makes it possible to effectively transfer beneficial genes through traditional breeding methods. Most species require the use of specialized genomic and chromosomal engineering technologies aimed at transforming their genetic diversity into a form compatible with traditional breeding [1,2].

In this light, the development and implementation of effective methods for the conservation and rational use of the gene pool of wild wheat relatives are key tasks of modern genetics and plant breeding. These methods play a crucial role in improving the characteristics of common wheat, including resistance to biotic and abiotic stresses. The genetic diversity of wild wheat relatives is a significant breeding resource, especially in terms of resistance to diseases and pests. Species belonging to the genera *Tritium*, *Aegilops*, *Agropiron*, *Secale*, *Haynaldia*, *Villosa*, and others possess valuable genes that can be introduced into wheat to increase its resistance to pathogens and pests. In addition, wild wheat relatives are sources of valuable agronomic traits such as frost resistance, drought tolerance, salt tolerance, and high protein quality. The introduction of these genes into cultivated wheat varieties can significantly increase their adaptive abilities and productivity in various climatic conditions [3,4].

The primary gene pool of common wheat includes species with homologous genomes: hexaploid forms (ancient varieties), cultivated tetraploid *T. turgidum* and its wild form *T. dicoccoides*, genome donor A: *T. monococcum* with varieties *boeoticum* and *urartu*, and genome donor D: *Aegilops tauschii*. Genes from the primary gene pool can be transferred by direct hybridization, homologous chromosome recombination, backcrossing, and selection [5].

Grain of the genus *Aegilops* L. is characterized by a high protein content—from 19 to 34% on a weight basis, exceeding soft wheat by a range of 1.5–2.5 times. The microsedimentation index (SDS) varies from 1.8 to 9.5 mL compared to 4.1 mL in wheat. The baking qualities of soft wheat grains are determined by a complex of genes localized in the D subgenome introduced into the wheat genome from *Aegilops tauschii*. In fact, wheat became a grain crop only after the genome of its ancestral form was combined with the genome of this species of Aegilops. Practically all polyploid species of Aegilops with the D genome also possess baking properties; synthetic amphidiploids obtained with the participation of this genome also possess them [6,7].

The main objective of this study was to characterize a new, genetically diverse source material for the breeding of common wheat based on botanical and morphological analysis, electrophoretic profiling of storage protein in spontaneous *Aegilops–*wheat hybrids, laser microscopy, and high-performance liquid chromatography tandem mass spectrometry (HPLC-MS/MS). Specifically, the following tasks were carried out:

- A botanical and morphological analysis of the ear of the Aegilops-wheat hybrids;
- Study and register Aegilops–wheat hybrids according to protein formulas;
- Analyze spontaneous fertile Aegilops–wheat hybrids using laser microscopy and tandem mass spectrometry.

## 2. Results

### 2.1. Morphological Characterization of Maternal Form and Spontaneous Hybrids

#### 2.1.1. Maternal Form *Aegilops trivialis* Migusch. Et Chak. k-1386 (Uzbekistan)

**Figure 1:**
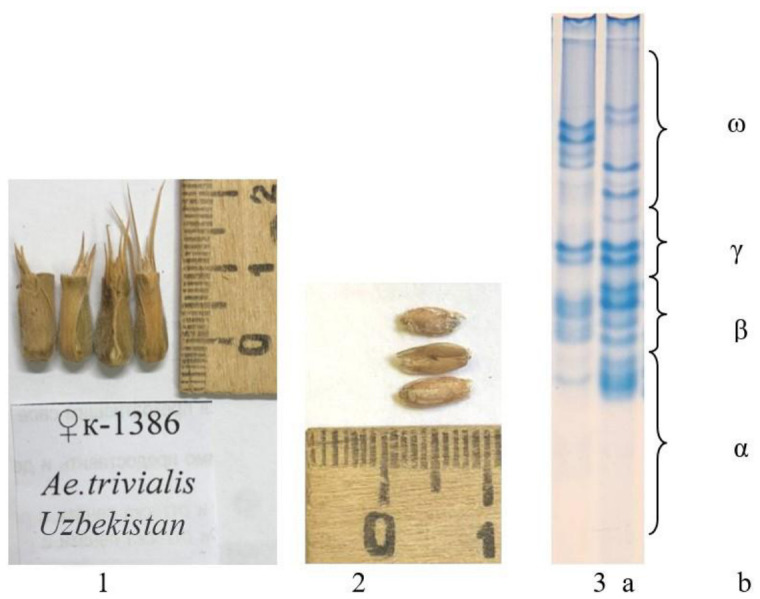
Maternal form k-1386: 1: ear, 2: seed, and 3: protein spectrum (a: 1386; b: St Caucasus).

The maternal form k-1386 (Figure 1) exhibits a distinctly spinous ear morphology characterized by wide, lamellar awns in the upper portion of the spike. These awns possess a pronounced median vein, a feature that distinguishes this taxon from other species with the genus *Aegilops*. The spike displays a knobby structure with weakly expressed spikelet swelling. The venation of the ear is represented by indistinct arc-shaped structures, which serve as a diagnostic feature of this morphotype. Notably, the analyzed form presents with pubescence, further enhancing its taxonomic uniqueness.

Protein formula of the maternal form k-1386: α 6 7|β 2 3 4|γ 2 3|ω 3 4 5 7 8.

#### 2.1.2. Spontaneous Hybrid k-4858 (*Ae. trivialis* k-1386 × *Triticum*)

**Figure 2:**
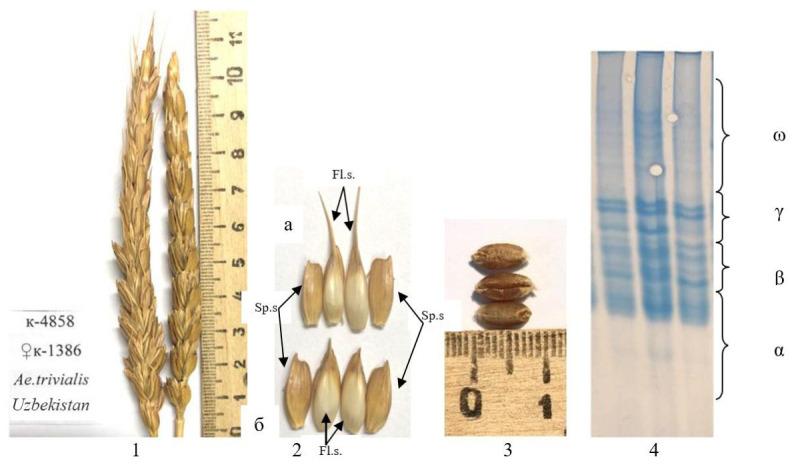
Spontaneous hybrid k-4858: 1: ear, 2: seed (Fl.s: flower scales; Sp.s.: spikelet scales), 3: seed, and 4: protein spectrum.

Ear morphology. The ear of the spontaneous hybrid k-4858 (Figure 2) is a typical sample of soft wheat with a number of specific morphological features. The color of the ear is light yellow, which is typical for many varieties of soft wheat. The ear has spinous appendages, but is spineless, which indicates that it belongs to the cultivated forms of wheat. The length of the ear is in the range of 8–10 cm, and the length of the spikelets varies from 8 to 11 mm. The spikelet scales are smooth and glossy, which indicates their high degree of development and adaptation to growing conditions. The ear is five-flowered, which is typical for many wheat varieties. The spikelet scales have a pronounced keel, which plays an important role in the process of pollination and grain formation. The shoulder of the spikelet scales is sloping, which is also a characteristic feature of soft wheat. There is no venation of the spikelet scales, which indicates its structural integrity and adaptability to various climatic conditions. The upper spikelets are sterile, which is an important aspect of the reproductive biology of this hybrid.

Grain morphology. The grain of the spontaneous hybrid k-4858 is naked, which indicates its high degree of maturity and adaptation to storage and processing conditions. The length of the grain is in the range of about 4–6 mm, which corresponds to the average grain size of soft wheat. The grain is almost vitreous, which indicates a high content of protein and other nutrients. The crest of the grain is pubescent, which is a characteristic feature of many wheat varieties.

The grain has an oval shape and a smooth surface, which indicates its structural integrity and adaptation to environmental conditions. The embryo shield is clear, undeformed, and pronounced, which indicates the good quality of the grain and its high viability. These morphological characteristics make the grain of the spontaneous hybrid k-4858 a valuable object for breeding research and practical application in agriculture.

Protein formula of spontaneous hybrid k-4858: α 5 6 7|β 2 3 4|γ 1 3 4|ω 3 4 5 6 7 8.

Protein analysis. The analysis of the protein formulas of the spontaneous hybrid (k-4858) and its maternal form (k-1386) revealed significant changes in the spectrum of protein components. Five new protein fractions were found in the alpha zone, and five new components were also detected in the beta zone. Changes were observed in the gamma zone, expressed in the appearance of 1, 4 new protein components. Two new proteins (components 6 and 9) were noted in the omega zone, while component 5, present in the maternal form, was absent from the spectrum of the spontaneous hybrid. These data indicate significant modifications of the proteome, which may be due to genetic and epigenetic factors, as well as environmental influences on the hybrid form.

#### 2.1.3. Spontaneous Hybrid k-4859 (*Ae. trivialis* × *Triticum*)

**Figure 3:**
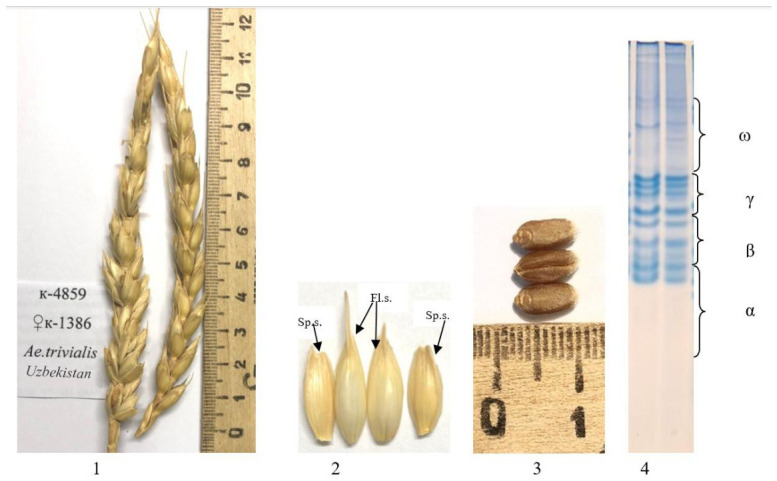
Spontaneous hybrid k-4859: 1: ear, 2: seed (Fl.s: flower scales; Sp.s.: spikelet scales), 3: seed, and 4: protein spectrum.

Ear morphology. The ear has a light-yellow color, which indicates a low intensity of photosynthetic processes. There are spinous processes on the ear, the length of which varies in the range of 6–10 mm, while they are characterized by a high degree of rigidity and sharpness, which may indicate adaptation to adverse climatic conditions. The length of the ear itself is in the range of 10–12 cm, and the length of the spikelets is in the range of 10–12 mm (Figure 3). Spikelet scales have a smooth, glossy surface, which may be due to their high resistance to mechanical damage and external influences. The spikelet scales have a semi-solid consistency, without pronounced neural lines, which may indicate a low content of lignin and other structural components. The keel on the spikelet scales is pronounced, which is an important morphological feature that affects the aerodynamic properties of the grain. The shoulder of the spikelet scales is beveled, which is typical for many cereal crops and can contribute to a more efficient grain distribution in the ear. The upper spikelets are usually sterile, which may be the result of genetic mutations or unfavorable growing conditions.

Grain morphology. The grain is a naked type, which indicates the absence of a film layer surrounding the grain. The length of the grain varies in the range of 4–8 mm, and it has a glassy texture, which indicates a high starch content. The crest of the grain is covered with pubescence, which can serve as an additional protective mechanism against pests and adverse weather conditions. The grain has an oval–elongated shape and a smooth surface, which contributes to its better storage and transportation. The embryo shield is clearly defined and undeformed, which indicates the high viability of the seed and its ability to germinate.

Protein formula of spontaneous hybrid k-4859 (option a): α 5 6 7|β 2 4 5|γ 2 3 4|ω 4 8 9.

Protein formula of spontaneous hybrid k-4859 (option b): α 5 6 7|β 2 4 5|γ 2 3 4|ω 2 4 8 9.

Protein analysis. A comparative analysis of the protein profiles of the spontaneous hybrid k-4859 and the maternal form k-1386 revealed significant differences in the component composition. In the alpha zone, the spontaneous hybrid has sub-component 7_1_, which is absent in the maternal form. In the beta zone, the parent component 3 disappeared, but a new component 5 appeared. In the gamma zone, the spontaneous hybrid has the presence of component 4, which is absent in the maternal form. In the omega zone of the spontaneous hybrid, a decrease in the number of components was revealed: components 3 and 5 present in the maternal form are absent in the spontaneous hybrid, while a new component 9 appeared.

Special attention should be paid to the omega zone, where component 2 exhibits unstable behavior, periodically appearing and disappearing. Additional electrophoretic studies are planned for a more detailed study of this issue and clarification of the component composition of the spontaneous hybrid k-4859.

#### 2.1.4. Spontaneous Hybrid k-4860 (*Ae. trivialis* k-1386 × *Triticum*)

**Figure 4:**
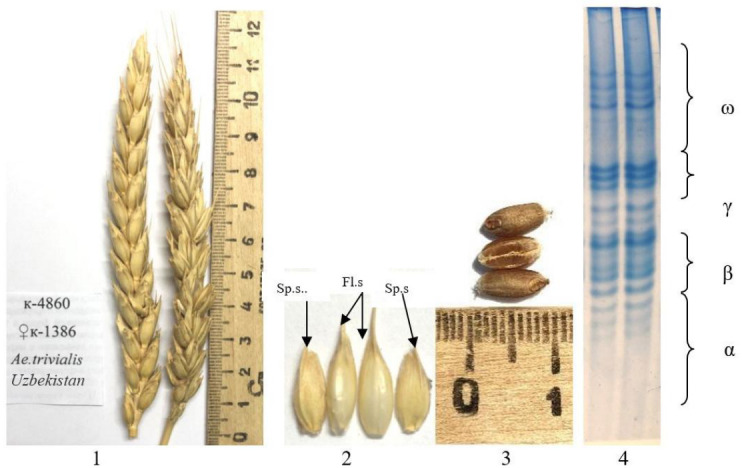
Spontaneous hybrid k-4860: 1: ear, 2: seed (Fl.s: flower scales; Sp.s.: spikelet scales), 3: seed, and 4: protein spectrum.

Ear morphology. The ear of the k-4860 hybrid is characterized by a light-yellow color and the presence of spinous processes. Its length varies in the range of 9–11 cm, and the spike has a size in the range of 10–12 mm. The spikelet scales are smooth and glossy, with a well-defined keel. The spinous processes are rigid, beak-shaped, and their length is in the range of 5–10 mm. The ear is five-flowered, with a pronounced sloping shoulder. The spikelet scales have high hardness and lack of nervousness. The upper spikelets are sterile (Figure 4).

Grain morphology. The grain of this hybrid belongs to the nudibranch type and has a length in the range of about 4–8 mm. It has a vitreous appearance and a pubescent crest. The grain has an oval–elongated shape and a smooth surface. The embryo shield is clearly defined and undeformed, which indicates a high degree of morphological integrity.

Protein formula of spontaneous hybrid k-4860: α 4 5 6|β 2 3 4 5|γ 2 3 4|ω 5 6 7 9.

Protein analysis. A comparative analysis of the protein profile of the spontaneous hybrid k-4860 and its parent form k-1386 revealed significant differences in the component composition. In the alpha zone, components 4 and 5 were identified, while component 7 was absent. In the beta zone, the changes were less pronounced, with only the addition of component 5. In the gamma zone, component 4 was observed. In the omega zone of the spontaneous hybrid, components 6 and 9 were identified, but components 3, 4, and 8 were absent.

#### 2.1.5. Spontaneous Hybrid k-4861 (*Ae. trivialis* k-1386 × *Triticum*)

**Figure 5:**
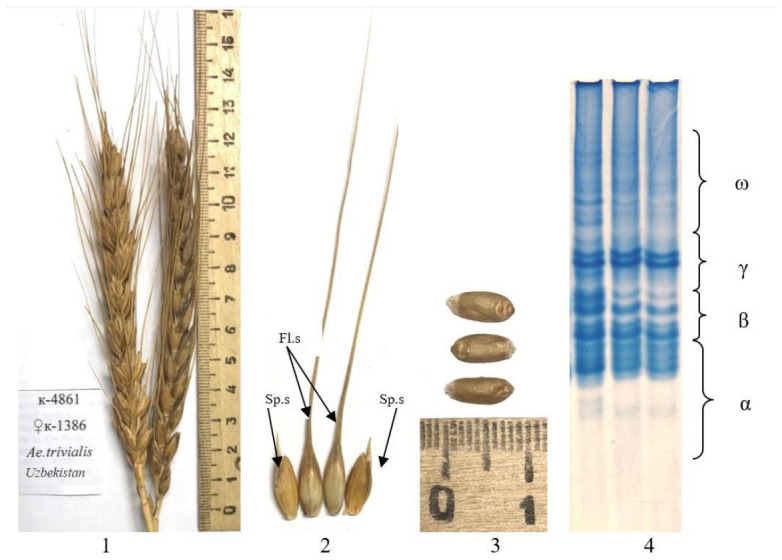
Spontaneous hybrid k-4861: 1: ear, 2: seed (Fl.s: flower scales; Sp.s.: spikelet scales), 3: seed, and 4: protein spectrum.

Ear morphology. The ear has a yellow color and a high degree of spinosity. Its length varies in the range of 9–11 cm, and the length of a single spike is in the range of 10–12 mm. The spikelet scales have a smooth and glossy surface. The extreme spikelet scales end in long awns, which are characterized by rigidity and divergent direction. The ear is five-flowered. The keel is clearly marked on the spikelet scales. The shoulder has a sloping shape. The spikelet scales are characterized by hardness and lack of nervousness. The upper spikelets are infertile (Figure 5).

Grain morphology. The grain of the nudibranch type is characterized by a length in the range of 4–6 mm and has a vitreous endosperm. The crest is covered with pubescence, which is a typical morphological feature of this species. The grain shape is oval, the surface is smooth, and the embryo’s carapace has a clear and undeformed structure, which indicates its high degree of morphological differentiation and functional maturity.

Protein formula of spontaneous hybrid k-4861: α 5 6 7|β 1|2 3 4|5 γ|2 3 4 5| ω 2 3 4 5 6 8 9.

Protein analysis. A comparative analysis of the protein formulas of the spontaneous hybrid k-4861 and its maternal form k-1386 revealed significant differences in the component composition.

A new component, identified as component 5, has been discovered in the alpha zone of the spontaneous k-4861 hybrid. Components 1 and 5 missing from the maternal form were identified in the beta zone. The gamma zone of the hybrid is characterized by the appearance of components 4 and 5 that were not registered in the original form. The omega zone shows a significant increase in its component composition, with the appearance of new components 2, 6, and 9 that are not present in the spectrum of the maternal form.

The analysis of the protein spectra of spontaneous hybrids presented in this work allows us to state that they demonstrate the variability of the component composition in all analyzed zones. These differences may be due to genetic modifications resulting from spontaneous hybridization, which leads to changes in the structure and functions of protein molecules.

**Figure 6:**
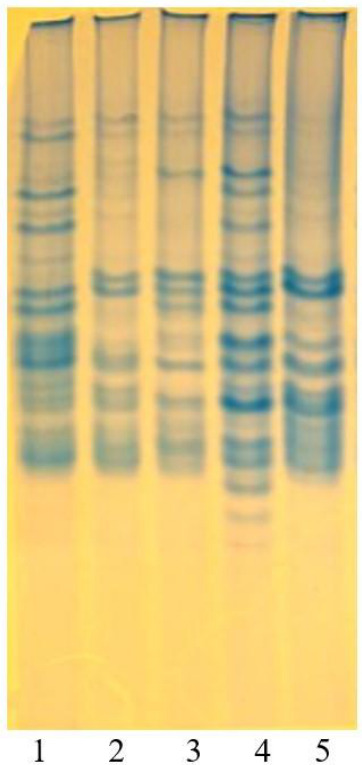
Spectra of spontaneous hybrids: 1: St. Caucasus (maternal form), 2: k-4858, 3: k-4859, 4: k-4860, and 5: k-4861.

Figure 6 clearly shows the differences between the protein spectra presented in this study. It should be noted that components 8 and 9 are present in the omega zone of the studied samples, which is an indicator of the presence of baking characteristics.

Of particular interest is the D-duplex of omega-89 gliadin components characteristic of the genome, which manifest themselves as slow in electrophoresis and are controlled by the 1D chromosome. This doublet, like the D genome as a whole, is associated with elastic gluten and the baking qualities of flour. All forms of hexaploid wheat and aegilops with the D genome and omega-89 gliadins have baking properties. It is assumed that this doublet marks homeologous chromosomes U and M, similar to chromosome 1D, which make the main contribution to the formation of prolamines in baking flour. The composition of gluten proteins in *Aegilops tauschii* Coss. and derivatives of polyploid wheat and aegilops species are characterized by a high content of insoluble gluten fraction P0 and a reduced content of the P2 fraction rich in gliadins. This results in a low gliadin:gluten ratio, similar to soft wheat [8].

The electrophoretic spectra of gliadin, like those of many other proteins, have a high degree of genotypic specificity. They demonstrate resistance to variations related to the year of reproduction and growing conditions of plants, which makes them indispensable as markers of genetic diversity. These spectra, like unique “fingerprints”, make it possible to differentiate varieties, biotypes, and lines at the molecular level, which leads to their widespread use in genetic research and breeding [9].

A comprehensive analysis was carried out as part of a study of spontaneous fertile Aegilops–wheat hybrids using modern analytical methods such as laser microscopy and tandem mass spectrometry.

### 2.2. Laser Microscopy Visualization of Phenolic Compounds

**Figure 7:**
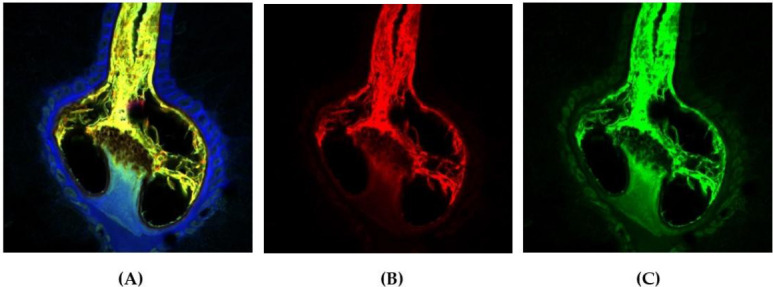
(**A**) Multispectral image of a longitudinal section of the seed variety spontaneous hybrid k-4858 *Ae. trivialis*, presented in all measured spectra. Excitation at 405 nm with emissions in the range of 400–475 nm (blue); excitation at 488 nm with emissions in the range of 500–545 nm (620–700 nm (red). (**B**) Presence of anthocyanin content spontaneous hybrid k-4858 *Ae. trivialis* (red color); (**C**) presence of flavonols spontaneous hybrid k-4858 *Ae. trivialis* (green color).

**Figure 8:**
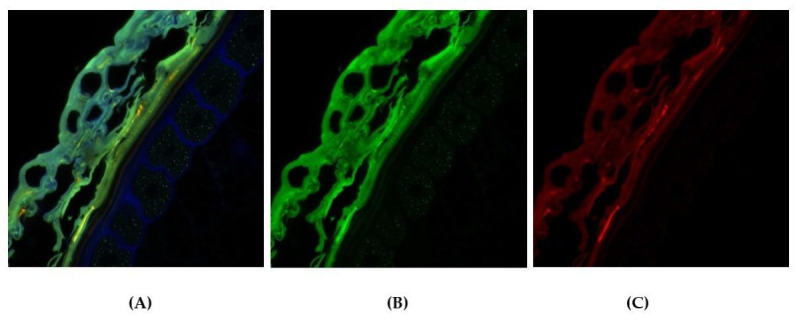
(**A**) Multispectral image of a transverse section of the seed variety spontaneous hybrid k-4859 *Ae. trivialis*, presented in all measured spectra. Excitation at 405 nm with emissions in the range of 400–475 nm (blue); excitation at 488 nm with emissions in the ranges of 500–545 nm (green) and 620–700 nm (red); (**B**) presence of flavonols spontaneous hybrid k-4859 *Ae. trivialis* (green color); (**C**) presence of anthocyanin content spontaneous hybrid k-4859 *Ae. trivialis* (red color).

**Figure 9:**
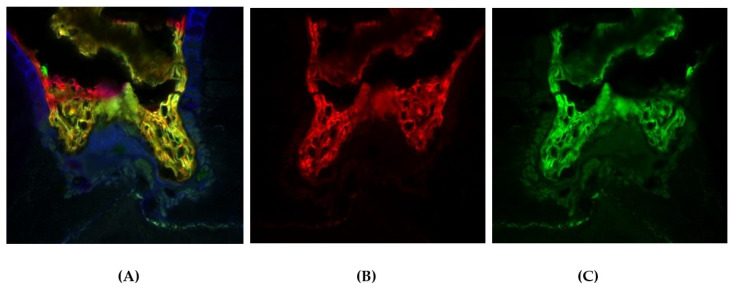
(**A**) Multispectral image of a longitudinal section of the seed variety spontaneous hybrid k-4860 *Ae. trivialis*, presented in all measured spectra. Excitation at 405 nm with emissions in the range of 400–475 nm (blue); excitation at 488 nm with emissions in the range of 500–545 nm (620–700 nm (red). (**B**) Presence of anthocyanin content spontaneous hybrid k-4860 *Ae. trivialis* (red color); (**C**) presence of flavonols spontaneous hybrid k-4860 *Ae. trivialis* (green color).

**Figure 10:**
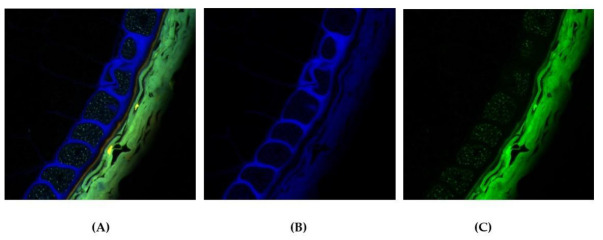
(**A**) Multispectral image of a transverse section of the seed variety spontaneous hybrid k-4861 *Ae. trivialis*, presented in all measured spectra. Excitation at 405 nm with emissions in the range of 400–475 nm (blue); excitation at 488 nm with emissions in the range of 500–545 nm (green). (**B**) Presence of hydroxycinnamic acids spontaneous hybrid k-4861 *Ae. trivialis* (blue color); (**C**) presence of flavonols spontaneous hybrid k-4861 *Ae. trivialis* (green color).

Visualization of the distribution of chemical components in Aegilops seeds by laser optical microscopy requires prior knowledge of the spectral characteristics of pure Aegilops components. The autofluorescence of various biochemical substances varies significantly, which makes it possible to identify them under a microscope. Studies have shown that transverse and longitudinal sections of Aegilops seeds exhibit intense fluorescence when using a laser confocal microscope, which indicates the presence of several substances with auto fluorescent properties in the studied samples (Figure 7, Figure 8, Figure 9 and Figure 10).

Figure 7A shows a multispectral longitudinal cross-sectional image of the spontaneous hybrid Aegilops k-4858, visualized over all measured spectral ranges. Figure 7B shows a spectral image in the red region of the spectrum, which indicates the presence of anthocyanins in the k-4858 sample. The intense spectral image in the green region of the spectrum (Figure 7C) indicates the presence of a flavonoid group in this sample.

Figure 8A shows a multispectral cross-sectional representation of the Aegilops k-4859 spontaneous hybrid, covering all measured spectral ranges. A comparative analysis of the spectral data presented in Figure 8C revealed a significantly less-pronounced presence of the anthocyanin group in the spontaneous hybrid Aegilops k-4859 compared with the sample k-4858 (Figure 7C).

Figure 10A shows a multispectral cross-sectional image of the spontaneous hybrid Aegilops k-4861, visualized over all measured spectral ranges. When comparing the multispectral image of the Aegilops k-4861 hybrid (Figure 10A) with the multispectral image of the Aegilops k-4859 hybrid (Figure 8A), a significant presence of hydroxycinnamic acids, visualized in the blue region of the spectrum, is observed in the walls of the aleurone layer of the k-4861 hybrid. At the same time, the presence of hydroxycinnamic acids in the k-4859 hybrid is expressed in a much smaller volume.

### 2.3. Metabolomic Profiling by LC-MS/MS

To characterize the phytochemical composition of the four hybrids, extracts were analyzed using high-performance liquid chromatography coupled with tandem mass spectrometry (HPLC-MS/MS). A total of one hundred twenty-eight chemically significant compounds were identified with a high degree of reliability, of which ninety-three compounds belong to the polyphenolic group. In addition, high-quality laser microscopy photographs were obtained, including longitudinal and transverse sections of the seed, as well as images in three different spectral ranges.

The HPLC conditions were optimized to obtain maximal resolution and signal within a minimal run time. Various chromatographic conditions such as mobile phase composition, injection volume, flow rate, column temperature, and gradient program were studied and optimized for the separation of polyphenol compounds. Different mobile phase compositions (ethanol–water, ethanol-0.1% (*v*/*v*) formic acid aqueous solution, acetonitrile–water, and acetonitrile −0.1% (*v*/*v*) formic acid aqueous solution) were tested in the gradient program at a 0.25 mL/flow rate.

As part of this study, our research team conducted an in-depth analysis of extracts of four spontaneous hybrids of *Ae. trivialis* (k-4858, k-4859, k-4860, k-4861) using tandem mass spectrometry. The purpose of this analysis was to determine the phytochemical profile of the studied samples as accurately as possible. The results showed that all four hybrids are characterized by a high content of biologically active compounds.

In the course of the study, one-hundred-and-one compounds of the polyphenolic class and thirty-five compounds belonging to other chemical groups were identified. Identification was carried out on the basis of a comparative analysis of retention indices, mass spectra, and MS fragmentation data compared with its own database created at the Laboratory of Biotechnology, Bioengineering, and Food Systems at the Far Eastern Federal University.

The proprietary database underlying this study is based on data obtained using various spectroscopic methods, including nuclear magnetic resonance (NMR), ultraviolet spectroscopy (UV), and magnetic resonance imaging (MRI), as well as information from specialized literature that is regularly updated and rechecked. The capture rate of the spectra was one spectrum per millisecond or two spectra per millisecond per mass spectrometer (MS). The data were collected using Windows software for BRUKER DALTONIKS (Munich, Germany). All experiments were repeated three times to ensure reproducibility of the results. The analysis was performed in a four-stage ion separation mode (MS/MS mode).

All previously identified compounds, including their molecular formulas, calculated and observed *m*/*z* ratios, MS/MS spectroscopy data, and comparative analysis results for spontaneous hybrids of *Ae. trivialis* (k-4858, k-4859, k-4860, k-4861), are presented in Appendix A, Table A1.

**Figure 11:**
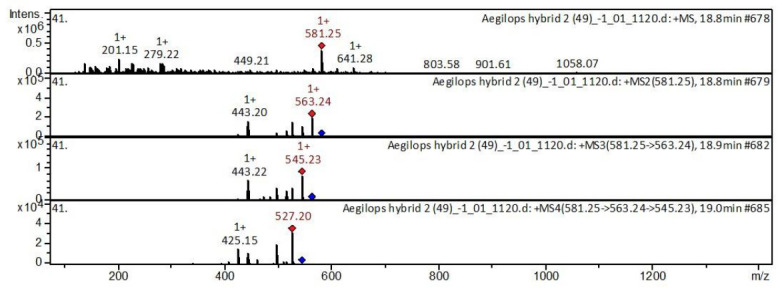
CID spectrum of luteolin 8-C-pentoside-6-C-hexoside from extracts of *Ae. trivialis*, *m*/*z* 581.25. At the top is an MS scan in the range of 100–1700 *m*/*z*; at the bottom are fragmentation spectra (from top to bottom): MS2 of the protonated luteolin 8-C-pentoside-6-C-hexoside ion (581.25 *m*/*z*, red diamond), MS3 of the fragment 581.25 → 563.24 *m*/*z*, and MS4 of the fragment 581.25 → 563.24 → 545.23 *m*/*z*.

**Figure 12:**
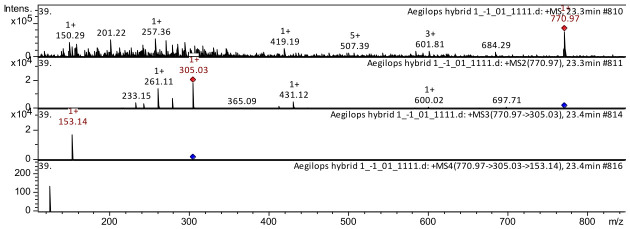
CID spectrum of apigenin-6-*C-β-*galactosyl-8-*C-β-*glycosyl-O-glycuronopyranoside from extracts of *Ae. trivialis*, *m*/*z* 770.97. At the top is an MS scan in the range of 100–1700 *m*/*z*; at the bottom are fragmentation spectra (from top to bottom): MS2 of the protonated apigenin-6-*C-β-*galactosyl-8-*C-β*-glycosyl-O-glycuronopyranoside ion (770.97 *m*/*z*, red diamond), MS3 of the fragment 770.97 → 305.03 *m*/*z*, and MS4 of the fragment 770.97 → 305.03 → 153.14 *m*/*z*.

**Figure 13:**
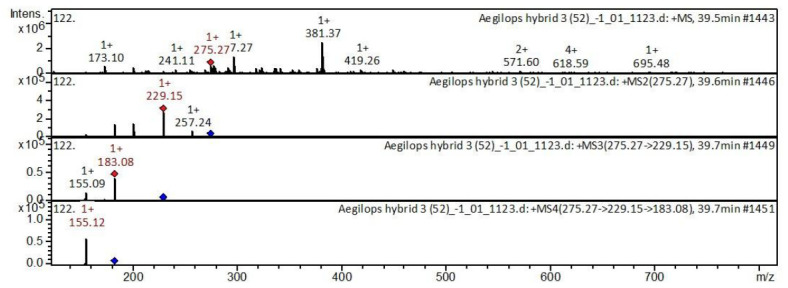
CID spectrum of afzelechin from extracts of *Ae. trivialis*, *m*/*z* 275.27. At the top is an MS scan in the range of 100–1700 *m*/*z*; at the bottom are fragmentation spectra (from top to bottom): MS2 of the protonated afzelechin ion (275.27 *m*/*z*, red diamond), MS3 of the fragment 275.27 → 229.15 *m*/*z*, and MS4 of the fragment 275.27 → 229.15 → 183.08 *m/z*.

**Figure 14:**
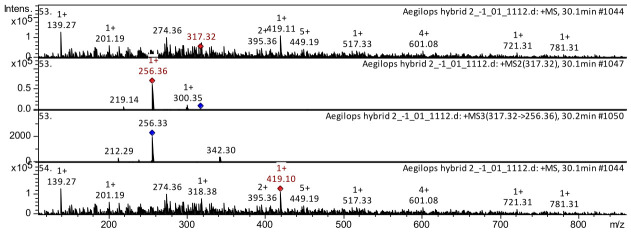
CID spectrum of isorhamnetin from extracts of *Ae. trivialis*, *m*/*z* 317.32. At the top is an MS scan in the range of 100–1700 *m*/*z*; at the bottom are fragmentation spectra (from top to bottom): MS2 of the protonated isorhamnetin ion (275.27 *m*/*z*, red diamond), MS3 of the fragment 317.32 → 256.36 *m*/*z*, and MS4 of the fragment 317.32 → 256.36 → 183.08 *m*/*z*.

The mass spectra of polyphenolic compounds, including flavones, flavan-3-ols, and flavonols, which were identified in spontaneous hybrids of *Ae. trivialis* (k-4858, k-4859, k-4860, k-4861), are presented below. These data are the result of a detailed analysis conducted using advanced mass spectrometry techniques, which made it possible to accurately determine the chemical composition of the samples under study. The inclusion of these spectra in scientific discourse allows us not only to deepen our understanding of the biochemical diversity of Aegilops, but also provides valuable information for further research in the field of genetics and evolution of cereals (Figure 11, Figure 12, Figure 13 and Figure 14). The flavone luteolin 8-C-pentoside-6-C-hexoside was found in extracts from Ae. trivialis (Figure 11).

The mass spectrum in the positive ion mode of luteolin 8-C-pentoside-6-C-hexoside from extracts of *Ae. trivialis* is shown in Figure 11. The [M + H]^+^ ion produced two fragment ions at *m*/*z* 563.24 and *m*/*z* 443.20. The fragment ion with *m*/*z* 563.24 yielded two daughter ions at *m*/*z* 545.23 and *m*/*z* 443.22. The daughter ion produced two fragment ions at *m*/*z* 527.20 and *m*/*z* 425.15. This bioactive substance was identified in mass spectrometric studies as luteolin 8-C-pentoside-6-C-hexoside in extracts of *Triticum aestivum* L. [10,11,12]; *Cynodon dactylon* [13].

The flavone apigenin-*6-C-β-*galactosyl-*8-C-β-*glycosyl-*O*-glycuronopyranoside was found in extracts from *Ae. trivialis* (Figure 12).

The mass spectrum in the positive ion mode of apigenin-*6-C-β-*galactosyl-*8-C-β-*glycosyl-*O*-glycuronopyranoside from extracts of *Ae. trivialis* is shown in Figure 12. The [M + H]^+^ ion produced seven fragment ions at *m*/*z* 697.71, *m*/*z* 600.02, *m*/*z* 431.12, *m*/*z* 365.09 *m*/*z* 305.03, *m*/*z* 261.11, and *m*/*z* 233.15. The fragment ion with *m*/*z* 305.03 yielded one daughter ion at *m*/*z* 153.14. This bioactive substance was identified in mass spectrometric studies as apigenin-*6-C-β-*galactosyl-*8-C-β-*glycosyl-*O*-glycuronopyranoside in extracts of *Triticum aestivum* [10].

The flavan-3-ol afzelechin was found in extracts from *Ae. trivialis* (Figure 13).

The mass spectrum in the positive ion mode of afzelechin from extracts of *Ae. trivialis* is shown in Figure 13. The [M + H]^+^ ion produced two fragment ions at *m*/*z* 229.15 and *m*/*z* 257.24. The fragment ion with *m*/*z* 229.15 yielded two daughter ions at *m*/*z* 183.08 and *m*/*z* 155.09. The daughter ion produced one fragment ion at *m*/*z* 155.12. This bioactive substance was identified in mass spectrometric studies as afzelechin in extracts of *Camellia kucha* [14]; *Pelargonium endlicherianum* [15]; *Rosa acicularis* [16]; *Loropetalum chinense* [17]; *Ventilago denticulata* [18].

The flavanol isorhamnetin was found in extracts of *Ae. trivialis* (Figure 14).

The mass spectrum in the positive ion mode of isorhamnetin from extracts of *Ae. trivialis* is shown in Figure 14. The [M + H]^+^ ion produced three fragment ions at *m*/*z* 300.35, *m*/*z* 256.36, and *m*/*z* 219.14. The fragment ion with *m*/*z* 256.36 yielded two daughter ions at *m*/*z* 256.33 and *m*/*z* 212.29. This bioactive substance was identified in mass spectrometric studies as isorhamnetin in extracts of *Inula viscosa* [19]; *Glycine soja* [20]; *Vaccinium macrocarpon* [21]; *Spondias purpurea* [22]; Andean blueberry [23]; *Lonicera caerulea* [24]; Eucalyptus [25]; Rapeseed petals [26]; *Ribes dikuscha*, *Ribes triste* [27]; *Stevia rebaudiana* [28]; *Syzygium aromaticum* [29]; *Rosa acicularis* [16]; Embelia [30]; *Rosa rugosa* [31]; *Rosmarinus officinalis* [32]; Propolis [33]; *Phoenix dactylifera* [34]; *Oligomeris linifolia* [35]; *Cyperus laevigatus* [36]; *Mint*; *Salvia* [37].

### 2.4. Comparative Analysis of Polyphenolic Compounds in Spontaneous Hybrids

**Figure 15:**
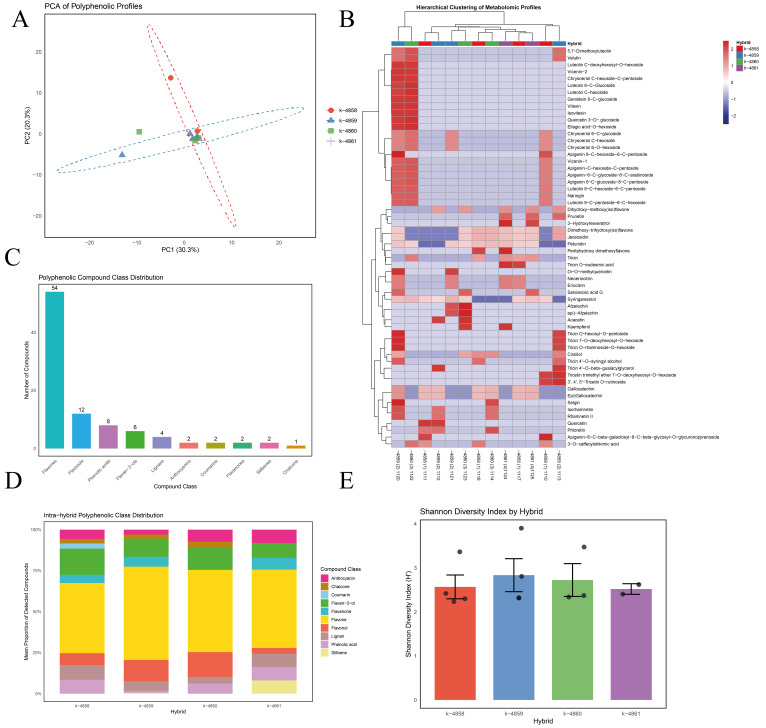
Comparative metabolomic analysis of four hybrids. (**A**) Principal component analysis, (**B**) hierarchical clustering heatmap, (**C**) number of compounds per class (total across all hybrids), and (**D**) stacked bar plot showing within hybrid compound variation. (**E**) Shannon diversity index.

Using HPLC-MS/MS, 93 polyphenolic compounds were found in the four spontaneous hybrids (k-4858, k-4859, k-4860, and k-4861). Principal component analysis showed that PC1 and PC2 explained 30.3% and 20.3%, respectively, cumulatively accounting for 50.6%. The PCA score plot revealed substantial overlap in metabolite profiles among the four hybrids, with no clear hybrid-specific clustering. Individual outlier replicates were observed for k-4858 (1116), k-4859 (1120), and k-4860 (1122), while the remaining replicates clustered together regardless of hybrid origin (Figure 15A). PCA loading analysis revealed that flavone C-glycosides, including vicenin-2, vitexin, isovitexin, apigenin derivatives, and luteolin derivatives, were the primary contributors to the negative PC1 axis (loadings ranging from −0.178 to −0.176). Apigenin and di-O-methylquercetin showed strong positive contributions to PC2 (loading = 0.218), while dimethoxy-trihydroxy(iso)flavone, jaceosidin, and petunidin contributed negatively to PC2 (loadings ranging from −0.121 to −0.118). This loading pattern indicates that the observed variation is driven primarily by differences in flavone glycoside abundance among individual replicates rather than by hybrid-specific chemical profiles. Similarly, the hierarchical clustering heatmap revealed distinct metabolite profiles among the four hybrids. The dendrogram showed moderate variation in metabolite profiles of hybrids. K-4858 and k-4860 grouped together for anthocyanins and some other compounds. Similarly, k-4858 and k-4859 were grouped together for several anthocyanins and chrysoeriol (Figure 15B). The compound class distribution revealed that flavones constituted the predominant polyphenolic class across the four hybrids (54 compounds), followed by flavonols (12 compounds), flavan-3-ols (6 compounds), phenolic acids (8 compounds), and others (Figure 15C). Stacked bar plot analysis demonstrated intra-hybrid variation in class composition: flavones were the most abundant class in all hybrids, with k-4859 showing the highest proportion, while phenolic acids, including hydroxycinnamic acid derivatives, were most prominent in k-4861 (Figure 15D). Shannon diversity index values ranged from 2.52 to 2.83 across the four hybrids, indicating moderate chemical diversity in all samples. Hybrid k-4859 exhibited the highest diversity (H′ = 2.83 ± 0.75), followed by k-4860 (H′ = 2.72 ± 0.65), k-4858 (H′ = 2.57 ± 0.54), and k-4861 (H′ = 2.52 ± 0.17). However, ANOVA revealed no significant differences in Shannon diversity among the hybrids (F = 0.177, *p* = 0.909) (Figure 15E). The observed differences (2.52 to 2.83) are likely due to random variation, not true biological differences.

**Figure 16:**
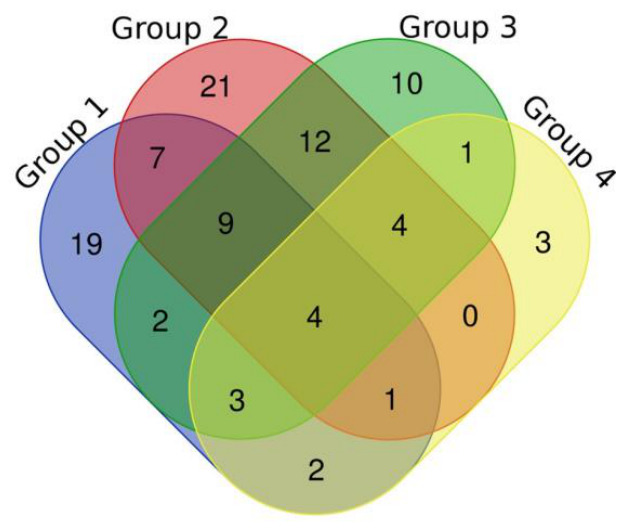
Venn diagram visualizing similarities and discrepancies between identified compounds of the polyphenolic class in four species of Aegilops.

A Venn diagram was created to show the similarities and differences in the polyphenolic profiles of the four hybrids (Figure 16). Each hybrid line’s species-specific metabolic characteristics and biochemical linkages are revealed through this graphical depiction of shared and distinct chemical ingredients. Four polyphenolic compounds were detected in all four hybrids: Petunidin (anthocyanidin), (Epi)Gallocatechin (flavan-3-ol), Syringaresinol (lignan), Gallocatechin (flavan-3-ol). These four substances appear in all hybrids, indicating that they constitute a conserved core metabolome that may be necessary for basic physiological processes or stress reactions in *Ae. derivatives* of *trivialis*. The hybrid k-4859 has the most distinct compounds (21), followed by k-4858 (19), k-4860 (10), and k-4861 (3). This suggests that the hybrids differ significantly in their metabolism, with k-4859 showing the most unique polyphenolic profile. Compared to other hybrid pairs, hybrids k-4859 and k-4860 had the greatest number of shared chemicals (12), indicating a closer metabolic similarity between these two lines. Instead of creating a consistent chemical profile, spontaneous hybridization with Triticum produced a variety of various metabolomic phenotypes, as seen by the significant number of unique chemicals in each hybrid (varying from 3 to 21). Together, these findings show that the genus Aegilops has a high degree of species-specific and hybrid-specific chemical compositions, with each spontaneous hybrid displaying a distinct polyphenolic fingerprint.

## 3. Discussion

The optical characteristics of plant tissues have a significant effect on the parameters of exciting light reaching the inner cell layers, as well as on the spectrum and intensity of fluorescent radiation emitted by plant cells and tissues. Absorption and re-emission of fluorescence inside the tissue can lead to a decrease in the observed fluorescent signal, which significantly complicates the use of spectroscopic methods for the analysis of intact plant samples. The transparency or opacity of plant tissues to excitatory radiation is due to the interaction of light with various structures such as epicuticular waxes and trichomes, as well as with cellular components, including phenolic compounds and photosynthetic pigments. These components, especially those located between the excitation source and the fluorescent detector, significantly affect the fluorescence intensity. As a result, the main source of fluorescent radiation in plant tissue is usually molecules localized in the epidermis and the upper layer of the mesophyll. The intensity of fluorescence from the underlying layers is significantly reduced due to the processes of light reabsorption. In addition, interactions with other molecules in the environment can lead to enhanced, modified, or suppressed fluorescence. All these factors must be taken into account when interpreting the fluorescent data obtained from plants [38].

When the terms “fluorescence” and “plants” are considered in context, botanists immediately associate them with the fluorescent properties of chlorophyll. These properties have been actively used in the last two decades as a powerful tool for non-invasive assessment of photosynthetic activity, physiological state of plants, and environmental impact. However, chlorophyll is only one of the many fluorophores present in plants. In addition to chlorophyll, many other fluorophores, such as phenolic compounds (anthocyanins, flavonoids, tannins, cinnamic acid), alkaloids, terpenoids, and porphyrins, also have the ability to fluoresce, and some of them emit light visible to the naked eye [39].

As already mentioned, the fluorescence intensity of plant compounds is influenced by many factors. It is noteworthy that when plants adapted to darkness are illuminated, there is a significant difference in the kinetics of chlorophyll and secondary metabolites fluorescence. Chlorophyll fluorescence exhibits complex induction kinetics known as the Kautsky curve, which reflects the overall efficiency of a photosystem after adapting to darkness. Since this kinetics changes under the influence of various stress factors, it has become an important tool for diagnosing the physiological state of plants. In contrast, the fluorescence of secondary metabolites is characterized by a more stable and specific response to light, which makes it a powerful tool for assessing specific stress conditions in plants. The analysis of specific blue–green (400–600 nm) and orange–red (600–800 nm) fluorescence of plants provides valuable information about their physiological state, diseases and abiotic stresses such as nutrient deficiency [40]. Fluorescent methods, as a rule, have high sensitivity, speed, and non-invasiveness. However, many of them require direct contact with or manipulation of tissues, which can lead to altered or distorted results. At the same time, laser-induced fluorescence is a non-invasive remote sensing method that allows analysis without collecting samples or direct contact with them [41].

It is assumed that the blue fluorescence observed in Aegilops seeds is due to the presence of phenolic compounds such as hydroxycinnamic acids [42]. In this class of compounds, ferulic acid is the main source of blue fluorescence, however, other compounds of this class, such as p-coumaric and caffeic acids, may also be associated with such fluorescence [43]. In addition, lignin, although associated with blue fluorescence in plant tissues [44], is either absent or present in insignificant amounts in the shells of Aegilops seeds, which suggests that the observed blue fluorescence is mainly due to hydroxycinnamic acids.

Aegilops seeds, in turn, are characterized by a high content of secondary metabolites such as flavonoids, stilbenes, coumarins, lignans, phenolic acids, and others. Flavonoids exhibit green autofluorescence at a wavelength ranging from 500 to 545 nm, which was confirmed by our results [45,46]. In addition, red fluorescence was detected due to the presence of anthocyanins and anthocyanidins [47,48]. These observations were further confirmed by mass spectrometry (MS), the results of which are presented in the relevant sections (Appendix A, Table A1).

Fluorescent flavonoids and their oxidation products, such as *Lignum nephriticum* (matlalin), have previously been described in the scientific literature [49]. It is important to note that they can also emit yellow and orange autofluorescence. Given the importance of flavonoids for the proper nutrition of humans and animals, as well as their role in plant resistance to biotic and abiotic stresses, the results obtained are important for their identification. In plants, flavonoids play a key role in auxin transfer, the development of root and shoot systems, the control of reactive oxygen species, pollination, the formation of symbiotic nodules, and perform protective functions [50]. Together with hydroxycinnamic acids and other weakly autofluorescent phenolic compounds, flavonoids are responsible for the fluorescence of the leaf epidermis [51,52].

Thus, in accordance with our previous studies of soybean and corn seeds [20,53], as well as data on Arabidopsis [54] and paprika [55], the use of laser optical microscopy to analyze the distribution of these compounds in various plant tissues is a valuable methodological approach.

## 4. Materials and Methods

### 4.1. Electrophoretic Analysis of Spontaneous Hybrids Ae. trivialis

In the framework of this study, a detailed analysis of four natural, spontaneous, fertile hybrids obtained by crossing aegilops and wheat, as well as their maternal form, the species *Aegilops trivialis* Migusch, was carried out by Et Chak. This analysis was carried out on the basis of the Dagestan Experimental Station, which is a branch of the Federal Research Center “N.I. Vavilov All-Russian Institute of Plant Genetic Resources”. Before starting laboratory studies, the hybrids were subjected to a multi-year check for stability and fertility, which made it possible to exclude possible phenotypic variations and ensure a representative sample.

The species *Aegilops trivialis* has the DDM genomic formula and belongs to the hexaploid species (2n = 42). In the systematic classification of the genus *Aegilops* L. proposed by E.F. Migushova, this species is included in the section *Vertebrata* (Zhuk) Kihara, which emphasizes its taxonomic significance and uniqueness.

A modified electrophoretic analysis technique developed at VIR was used to analyze the reserve proteins of gliadins isolated from individual grains of the studied samples. This technique, adapted for cereal crops, includes several key steps [9]. Gliadin extraction was carried out using a 6 M urea solution, which made it possible to effectively extract protein from pre-crushed grains. Electrophoresis was performed in plates of 6.5% polyacrylamide gel (PAAG) in an acetate buffer with a pH of 3.1. In the first hours, electrophoresis was performed at a current of 20 mA per plate and a voltage of 300 V, and in the next four hours, with an increase in current to 40 mA and voltage to 580 V.

Upon completion of electrophoretic separation, the plates were fixed in a solution containing 0.25% aqueous Coomassie G-250 and 12.5% trichloroacetic acid (TAC). The resulting-colored plates were photographed, and the spectra were recorded in the form of “protein formulas” reflecting the component composition of each spectral zone. At the same time, parameters such as the mobility of components, their intensity, and the presence of sub-components designated by indexes 1 and 2 were taken into account. For standardization and unification of data, the well-studied spectrum of gliadin of the Caucasus wheat variety was used as a reference spectrum, which ensured comparability of the results and their objectivity.

The minimum standard sample for analysis included three grains for both spontaneous hybrids and maternal forms. A total of 45 individual grains were analyzed, which allowed us to obtain representative data and draw reasonable conclusions about the genetic characteristics of the studied samples.

### 4.2. Chemicals and Reagents

All chemicals used in this study were of analytical grade. High-performance liquid chromatography (HPLC)-grade acetonitrile was purchased from Fisher Scientific (Loughborough, United Kingdom). Mass-spectrometry (MS)-grade formic acid was purchased from Sigma-Aldrich (Darmstadt, Germany). Ultra-pure water was prepared by using a SIEMENS ULTRA clear (SIEMENS water technologies, Berlin, Germany).

### 4.3. Fractional Maceration

Fractional maceration technique was used to obtain highly concentrated extracts [56]. Aqueous ethanol (80%) was used for extraction. From 150 g of the *Ae. trivialis*, 20 g of *Ae. trivialis* of each variety were randomly selected for maceration. The total amount of the extractant (aqueous ethanol 95%) was divided into 3 parts, and the parts of plant were consistently infused with the first, second, and third parts. The infusion of each part of the extractant lasted seven days at room temperature. Three replicates of the extraction process were carried out on each plant sample. The extract was filtered through Whatman filter paper. The filtrates were diluted with acetonitrile to final working concentration for analysis.

### 4.4. Liquid Chromatography

High-performance liquid chromatography was performed using a Shimadzu LC-20 Prominence HPLC (Shimadzu, Kyoto, Japan) equipped with a UV sensor and a C18 silica reverse phase column (4.6 × 150 mm, particle size: 2.7 μm) to perform the separation of multicomponent mixtures. The gradient elution program with two mobile phases (A, deionized water; B, acetonitrile with formic acid 0.1% *v*/*v*) was as follows: 0–2 min, 0% B; 2–50 min, 0–100% B; control washing 50–60 min 100% B. The entire HPLC analysis was performed with a UV–vis detector SPD-20A (Shimadzu, Kyoto, Japan) at a wavelength of 230 nm for identification of compounds; the temperature was 25 °C, and the total flow rate 0.25 mL min^−1^. The injection volume was 10 µL. Additionally, liquid chromatography was combined with a mass spectrometric ion trap to identify compounds.

### 4.5. Mass Spectrometry

Mass spectrometry analysis was performed on an ion trap amaZon SL (BRUKER DALTONIKS, Munich, Germany) equipped with an ESI source in positive and negative ion modes. The optimized parameters were obtained as follows: ionization source temperature: 70 °C, gas flow: 4 L/min, nebulizer gas (atomizer): 7.3 psi, capillary voltage: 4500 V, end plate bend voltage: 1500 V, fragmentary: 280 V, and collision energy: 60 eV. An ion trap was used in the scan range of *m*/*z* 100 −1.700 for MS and MS/MS. The chemical constituents were identified by comparing their retention index, mass spectra, and MS fragmentation with an in-house self-built database (Biotechnology, Bioengineering and Food Systems Laboratory, Far-Eastern Federal University, Vladivostok, Russia). The in-house self-built database is based on data from other spectroscopic techniques, such as nuclear magnetic resonance, ultraviolet spectroscopy, and MS, as well as data from the literature that is continuously updated and revised. The capture rate was one spectrum/s for MS and two spectrum/s for MS/MS. Data acquisition was controlled by Windows software for BRUKER DALTONIKS. All experiments were repeated three times. A four-stage ion separation mode (MS/MS mode) was implemented. The work is supported financially by Program Priority-2030 of Tomsk State University.

### 4.6. Data Analysis

All statistical analyses were performed in R (v 4.5.3; https://www.r-project.org/) with the following packages: “readxl” for data import, “ggplot2” for principal component analysis (PCA) and bar plots, “pheatmap” for hierarchical clustering heatmaps, “dplyr” and “tidyr” for data manipulation. Raw peak area data were log-transformed. PCA was performed on the log-transformed data with mean-centering and scaling. Hierarchical clustering was conducted using Euclidean distance and Ward’s method. Compound class distribution was analyzed using bar plots, stacked bar plots, and heatmaps. Shannon diversity index was calculated in R using “vegan” package.

## 5. Conclusions

In conclusion, it should be emphasized that in the course of this study, we were able to identify significant differences between the studied spontaneous hybrids *Aegilops trivialis*, as well as establish specific variations in the protein spectra of these hybrids compared with their parent form k-1386.

The presented spontaneous Aegilops–wheat hybrids will serve as valuable material for further research aimed at identifying their paternal form. However, it should be noted that the use of these hybrids in future breeding programs seems appropriate from the point of view of expanding the gene pool of the genus *Triticum* L. and creating new heterogeneous forms, which is important for agrobiotechnology and genetics of cultivated plants.

## Data Availability

The original contributions presented in this study are included in the article. Further inquiries can be directed to the corresponding authors.

## Appendix A

**Table A1:** Chemical compounds identified from the extracts of four spontaneous hybrids of *Ae. trivialis* (k-4858, k-4859, k-4860, k-4861) in positive and negative ionization modes by HPLC-ion trap-MS/MS.

	Class of Compounds	Identification	Formula	Calculated Mass	Observed Mass [M − H]−	Observed Mass [M + H]+	MS/MS Stage 2 Fragmentation	MS/MS Stage 3 Fragmentation	MS/MS Stage 4 Fragmentation	References
		**Polyphenols**								
1	Flavone	**Pinocembrin** [(+)-Pinocembrin; Pihyrochrysin; (2S)-Pinocembrin]	C_15_H_12_O_4_	256.25		285	257	201; 219; 131		*Punica granatum* [57]; *Rosa amblyotis* [16]; *Loropetalum chinense* [17]; *Propolis* [33]
2	Flavone	**Apigenin** [5,7-Dixydroxy-2-(40Hydroxyphenyl)-4H-Chromen-4-One]	C_15_H_10_O_5_	270.23		271	254; 236; 218; 187; 150	200; 172; 132		*Triticum aestivum* L. [10,12]; Propolis [33]
3	Flavone	**Prunetin** [Padmakastein]	C_16_H_12_O_5_	284.26		285	257	173; 237; 111	117	*Triticum aestivum* [58]; *Rosa rugosa* [31]
4	Flavone	**Dihydroxy-methoxy(iso)flavone**	C_16_H_12_O_5_	284.26		285	270; 257			Propolis [33]
5	Flavone	**Acacetin**	C_16_H_12_O_5_	284.26		285	270; 257; 201; 155	215; 181		*Triticum aestivum* [58]; *Rosa acicularis* [16]; *Ribes dikuscha*, *Ribes triste* [27]; Propolis [33]
6	Flavone	**Chrysoeriol** [Chryseriol]	C_16_H_12_O_6_	300.26		301	283	268; 184	145	*Rhus coriaria* [59]; *Triticum aestivum* L. [12]; *Rosa rugosa* [16]; *Loropetalum chinense* [17]; *Propolis* [33]
7	Flavone	**5,7-Dimethoxyluteolin**	C_17_H_14_O_6_	314.28		315	158; 239	130		*Rosa rugosa*; *Rosa davurica* [60]; *Glycine soja* [20]; *Syzygium aromaticum* [29]
8	Flavone	**Velutin** [Flavoyadorigenin B; Luteolin 7,3′-dimethyl ether]	C_17_H_14_O_6_	314.28		315	158	130		*Triticum aestivum* [58]
9	Flavone	**Tricin** [5,7,4′-trihydroxy-3′,5′-dimetoxyflavone]	C_17_H_14_O_7_	330.28	329		314	299	271	*Triticum aestivum* [10,12,58]
10	Flavone	**Cirsiliol**	C_17_H_14_O_7_	330.28		332	314	264	218	*Juglans mandshurica* [61]; *Inula viscosa* [19]
11	Flavone	**Hydroxy trimethoxyflavone**	C_18_H_16_O_6_	328.31	327		314; 229	299	271; 227	*G. linguiforme*; *F. pottsii* [62]; Propolis [33]
12	Flavone	**Dimethoxy-trihydroxy(iso)flavone**	C_17_H_14_O_7_	330.28	329		314	299	271	*Jatropha* [63]; Propolis [33]
13	Flavone	**Jaceosidin** [5,7,4′-trihydroxy-6′,5′-dimethoxyflavone]	C_17_H_14_O_7_	330.28	329		314	299	271	Mentha [64]
14	Flavone	**Di-*O*-methylquercetin**	C_17_H_14_O_7_	330.28	329		311; 229	211		*Triticum aestivum* [58]; *Loropetalum chinense* [17]
15	Flavone	**Pentahydroxy dimethoxyflavone**	C_17_H_14_O_9_	362.28		363	300; 256; 146	146	116	*G. linguiforme*; *F. pottsii* [62]; Chinese herbal formula Jian-Pi-Yi-Shen pill [65]
16	Flavone	**Tricin O-glycerol**	C_20_H_20_O_9_	404.36		405	331	315	270	*Eleocharis dulcis* [66]
17	Flavone	**Vitexin** [Apigenin 8-C-Glucoside]	C_21_H_20_O_10_	432.37		433	415; 367; 313; 283	337; 283	309; 281	*Triticum aestivum* L. [10,58,67]
18	Flavone	**Isovitexin** [Saponaretin; Homovitexin; Apigenin-6-C-Glucoside]	C_21_H_20_O_10_	432.37		433	415; 367; 313; 283	337; 283	309; 281	Chilean currants [68]; *Triticum aestivum* L. [12]; *Rhus coriaria* [59]
19	Flavone	**Genistein 8-C-glucoside**	C_21_H_20_O_10_	432.37		433	415; 367; 313; 283	337; 283	309; 281	Mexican lupine species [69]
20	Flavone	**Luteolin 8-C-Glucoside** [Orientin; Lutexin]	C_21_H_20_O_11_	448.37	447		357; 429	339	311; 175	*Fagopyrum esculentum* [70]; *Triticum aestivum* L. [10,11,67]
21	Flavone	**Luteolin C-hexoside** [Isoorientin; Homoorientin]	C_21_H_20_O_11_	448.37	447		357; 429	339; 297	311; 225	*Triticum aestivum* L. [11,12,58]; *Fagopyrum esculentum* [70]
22	Flavone	**Chrysoeriol *C*-hexoside**	C_22_H_22_O_11_	462.40		463	445; 397; 343	367; 313	339; 311	*Triticum aestivum* [71]
23	Flavone	**Chrysoeriol 6-*O*-hexoside**	C_22_H_22_O_11_	462.40		463	445; 397; 343; 313	313	313; 298	*Triticum aestivum* L. [11,12,71]
24	Flavone	**Chrysoeriol 6-C-glucoside** [Isoscoparin; Isoscoparine]	C_22_H_22_O_11_	462.40		463	445; 397; 343	367; 313	339; 311	*Triticum aestivum* L. [11,12,71]; *P. aculeata* [72]
25	Flavone	**Tricin 7-*C-*hexoside**	C_23_H_24_O_12_	492.42		493	331	316	315	*Triticum aestivum* L. [12]
26	Flavone	**Tricin O-hexoside**	C_23_H_24_O_12_	492.42		493	331	316	315	*Triticum aestivum* L. [11,73]
27	Flavone	**Tricin 5-O-hexoside**	C_23_H_24_O_12_	492.42		493	331	316	315	*Triticum aestivum* [58]
28	Flavone	**Tricin 4′-*O*-syringyl alcohol**	C_26_H_24_O_10_	496.46		497	331	315	270	*Triticum aestivum* [58]
29	Flavone	**Tricin trimethyl ether 7-O-hexoside**	C_24_H_26_O_12_	506.45		507	345; 199	284	255	*Triticum aestivum* L. [12]
30	Flavone	**Dihydroxy-trimethoxyflavone-*O*-hexoside**	C_24_H_26_O_12_	506.45		507	345; 199	284	255	Citrus species [74]; *Glycine soja* [20]
31	Flavone	**Tricin O-eudesmic acid**	C_27_H_24_O_11_	524.47		525	331	315	270	*Triticum aestivum* [58]
32	Flavone	**Tricin 4′*-O-beta*-guaiacylglycerol**	C_24_H_30_O_14_	526.48		527	331	315; 270	270	*Loropetalum chinense* [17]; *Eleocharis dulcis* [66]
33	Flavone	**Apigenin 8-C-hexoside-6-C-pentoside**	C_26_H_28_O_14_	564.49		565	547; 427	529; 427	511; 409; 325	*Triticum aestivum* L. [10,11,71]; *Cynodon dactylon* [13]
34	Flavone	**Apigenin-6-C-glycoside-8-C-arabinoside**	C_26_H_28_O_14_	564.49		565	547; 427	529; 427	511; 481; 409	*Camellia kucha* [14]
35	Flavone	**Apigenin 6-C-glucoside-8-C-pentoside**	C_26_H_28_O_14_	564.49		565	547; 427	529; 427	511; 409; 325	*Artemisia annua* [75]
36	Flavone	**Apigenin-C-hexoside-C-pentoside**	C_26_H_28_O_14_	564.49		565	511; 427; 251	511; 427; 295	427; 391; 313	*Triticum aestivum* [73,76]
37	Flavone	**Vicenin-1** [Apigenin 8-*C*-glucoside 6-*C*-xyloside]	C_26_H_28_O_14_	564.49		565	547; 427	529; 427	511; 409; 325	Jatropha [63]
38	Flavone	**Naringin** [Naringenin 7-O-Neohesperidoside]	C_27_H_32_O_14_	580.53		581	563; 443	545; 443	527; 425	Exocarpium Citri Grandis [77]; *G. linguiforme*; *F. pottsii* [62]; Mentha [64]
39	Flavone	**Luteolin 8-*C*-pentoside-6-*C*-hexoside**	C_26_H_28_O_15_	580.49		581	545; 443	527; 425	443	*Triticum aestivum* [10,11,76]; *Cynodon dactylon* [13]
40	Flavone	**Luteolin 8-*C*-hexoside-6*-C*-pentoside**	C_26_H_28_O_15_	580.49		581	563; 443	545; 443	527; 425	*Triticum aestivum* [11,71,73,76]; Jatropha [63]
41	Flavone	**Vicenin-2** [Apigenin-6,8-Di-*C*-Glucoside]	C_27_H_30_O_15_	594.51		595	577; 457	559; 457	541; 439	Lemon, Passion fruits [78]; *Triticum aestivum* L. [10,58,76]; *P. aculeata* [72]; *Exocarpium Citri Grandis* [77]
42	Flavone	**Chrysoeriol C-hexoside-C-pentoside**	C_27_H_30_O_15_	594.51		595	577; 457; 415; 367; 313	559; 511; 457; 415; 349	511; 481	*Triticum aestivum* [71,73]
43	Flavone	**Luteolin *C*-deoxyhexosyl*-O*-hexoside**	C_27_H_30_O_15_	594.51		595	577; 457	559; 457	541; 439	*Triticum aestivum* L. [12]
44	Flavone	**methoxylated O-pentosyl isoorientin**	C_28_H_32_O_15_	608.54		609	463; 445; 397; 343	445; 427; 397; 313	367; 313	*Triticum aestivum* L. [11,12]
45	Flavone	**Chrysoeriol O-deoxyhexoside-C-hexoside**	C_28_H_32_O_15_	608.54		609	463; 445; 397; 343	445; 427; 397; 313	367; 313	*Triticum aestivum* [12,71]
46	Flavone	**Chrysoeriol C-hexosyl-O-rhamnoside**	C_28_H_32_O_15_	608.54		609	463; 445; 397; 343	445; 427; 397; 313	367; 313	*Triticum aestivum* [58,73]
47	Flavone	**Chrysoeriol O-hexoside C-hexoside**	C_28_H_32_O_16_	624.54		625	463; 505; 607	445; 397	397	*Triticum aestivum* L. [12,58,73]
48	Flavone	**Chrysoeriol O-hexosyl- O-hexoside**	C_28_H_32_O_16_	624.54		625	463; 505; 607	445; 397	397	*Triticum aestivum* [58]
49	Flavone	**Tricin 7-O-deoxyhexosyl-O-hexoside**	C_29_H_34_O_16_	638.57		639	493; 331	331	316	*Triticum aestivum* L. [12,71]
50	Flavone	**Tricin O-rhamnoside-O-hexoside**	C_29_H_34_O_16_	638.57		639	493; 331	331	315; 270	*Triticum aestivum* L. [73]
51	Flavone	**Tricin O-hexosyl-O-pentoside**	C_29_H_34_O_16_	638.57		639	493; 331	331	316	*Triticum aestivum* L. [11]
52	Flavone	**Tricetin trimethyl ether 7-O-deoxyhexosyl-O-hexoside**	C_30_H_36_O_16_	652.59		653	507; 345	345	284	*Triticum aestivum* L. [12]
53	Flavone	**3′, 4′, 5′-Tricetin O-rutinoside**	C_30_H_36_O_16_	652.59		653	507; 345	345	284	*Triticum aestivum* [58]
54	Flavone	**Apigenin-6-C-beta-galactosyl-8-C-beta-glycosyl-O-glycuronopyranoside**	C_33_H_38_O_21_	770.64		771	697; 600; 431; 365; 305	153		*Triticum aestivum* L. [10]
55	Flavonol	**Kaempferol**	C_15_H_10_O_6_	286.23	285		257; 185; 117	117		Andean blueberry [23]; Rapeseed petals [26]; *Oligomeris linifolia* [35]; *Rhus coriaria* [59]; *Juglans mandshurica* [61]
56	Flavonol	**Quercetin**	C_15_H_10_O_7_	302.23						*Inula graveolens* [79]; *Inula viscosa* [19]; Eucalyptus [25]; Propolis [33]; *Rhus coriaria* [59]; *Juglans mandshurica* [61]
57	Flavonol	**Kumatakenin** [Kaempferol 3,7-dimethyl ether]	C_17_H_14_O_6_	314.28		315	287	187; 241		*Triticum aestivum* [58]
58	Flavonol	**Selgin** [Selagin; 3-O-Methyltricetin; Tricetin ′3-O-methyl ether]	C_16_H_12_O_7_	316.26		317	299; 217; 147	252	196	*Triticum aestivum* [80]
59	Flavonol	**Isorhamnetin** [Isorhamnetol; Quercetin 3′-Methyl ether; 3-Methylquercetin]	C_16_H_12_O_7_	316.26		317	302	274; 190	274	*Inula viscosa* [19]; Embelia [30]; *Rosa rugosa* [31]; *Rosmarinus officinalis* [32]; Propolis [33]; *Phoenix dactylifera* [34]; *Oligomeris linifolia* [35]; *Cyperus laevigatus* [36]; Mint; Salvia [37]
60	Flavonol	**Rhamnetin II**	C_16_H_12_O_7_	316.26		317	301	287; 256	159	*Polygala sibirica* [81]; *Inula graveolens* [79]; *Rosa acicularis* [16]; *Spondias purpurea* [22]; *Ribes dikuscha*, *Ribes triste* [27]; *Syzygium aromaticum* [29]; *Rhus coriaria* [59]
61	Flavonol	**Myricetin**	C_15_H_10_O_8_	318.23		319	317; 289; 219	301; 261; 191	241	*Inula graveolens* [79]; *Rosa acicularis* [16]; *Juglans mandshurica* [61]; *G. linguiforme*; *F. pottsii* [62]
62	Flavonol	**Di-*O*-methylquercetin**	C_17_H_14_O_7_	330.28	329		229	210	209	*Triticum aestivum* [58]
63	Flavonol	**Kaempferol-*8-C-beta-D-*glucopyranoside**	C_21_H_20_O_11_	448.37	447		357; 429	339	311; 175	*P. aculeata* [72]
64	Flavonol	**Quercetin 3-O-glucoside** [Isoquercetin; Isoquercitrin; Hirsutrin]	C_21_H_20_O_12_	464.37		465	303; 235	235	218; 138	*Spondias purpurea* [22]; Andean blueberry [23]; Rapeseed petals [26]; Embelia [30]; *Rhus coriaria* [59]; *Juglans mandshurica* [61]
65	Flavonol	**Myricetin-3-*O*-galactoside**	C_21_H_20_O_13_	480.37		481	319; 197	179	161; 133	*Rosa acicularis* [16]; *Loropetalum chinense* [17]; *Vaccinium macrocarpon* [21]; *Ribes dikuscha*, *Ribes triste* [27]; *Juglans mandshurica* [61]
66	Flavonol	**Myricetin-3-*O*-glucoside**	C_21_H_20_O_13_	480.37		481	319	197; 179	161; 133	*Triticum aestivum* [82]; *Camellia kucha* [14]; *Rosa acicularis* [16]; *Ribes dikuscha*, *Ribes triste* [27]; *Rhus coriaria* [59]
67	Flavanone	**Neoeriocitrin**	C_27_H_32_O_15_	596.53		597	331	315	270	Exocarpium Citri Grandis [77]
68	Flavanone	**Eriocitrin** [Eriodictoyl-7-O-rutinoside]	C_27_H_32_O_15_	596.53						Lemon, Passion fruits [78]; *Triticum aestivum* [58]; *G. linguiforme*; *F. pottsii* [62]; Exocarpium Citri Grandis [77]
69	Flavan-3-ol	**Afzelechin**	C_15_H_14_O_5_	274.26		275	257; 229	183; 155	155	*Camellia kucha* [14]; *Pelargonium endlicherianum* [15]
70	Flavan-3-ol	**(epi)-Afzelechin**	C_15_H_14_O_5_	274.26		275	257	183		*Glycine soja* [20]; *F. glaucescens*; *G. linguiforme*; *F. pottsii* [62]
71	Flavan-3-ol	**(epi)-Catechin**	C_15_H_14_O_6_	290.26		292	273; 191	255	239; 169	Black soja [83]; Eucalyptus [25]; Jatropha [63]
72	Flavan-3-ol	**Gallocatechin** [+(−)Gallocatechin]	C_15_H_14_O_7_	306.26		307	261	243	173	*Carpinus betulus* [84]; Embelia [30]; *G. linguiforme*; *F. pottsii* [62]
73	Flavan-3-ol	**(Epi)Gallocatechin**	C_15_H_14_O_7_	306.26		307	289	262; 194; 151		*Carpinus betulus* [84]; *G. linguiforme*; *F. pottsii* [62]; Chilean currants [68]
74	Flavan-3-ol	**Epiafzelechin derivative**	C_18_H_16_O_10_	392.31		393	375; 245	357; 222		*Ribes fragrans* [85]; *Lonicera caerulea* [24]
75	Anthocyanin	**Petunidin**	C_16_H_13_O_7+_	317.27		318	300	282	264	Wines [86]; *G. linguiforme*; *C. edulis*; *F. pottsii* [62]
76	Anthocyanin	**Petunidin 3-O-glucoside-acetaldehyde**	C_24_H_23_O_12_	503.43		503	341; 318; 271; 219; 185	279; 219		Wines [86]
77	Phenolic acid	**p-Coumaric acid** [4-Hydroxycinnamic acid]	C_9_H_8_O_3_	164.15		165	149; 119	119		Rapeseed petals [26]; *Cyperus laevigatus* [36]; *Rhus coriaria* [59]; *Juglans mandshurica* [61]
78	Phenolic acid	**Methylgallic acid** [Methyl gallate; Methyl 3,4,5-trihydroxybenzoate]	C_8_H_8_O_5_	184.14		185	153	123		Papaya, Lemon, Passion fruits [78]; *Rosa acicularis* [16]; *Lonicera caerulea* [24]; *Ribes dikuscha*, *Ribes triste* [27]; *Rhus coriaria* [59]
79	Phenolic acid	**Syringic acid** [Benzoic acid; Cedar acid]	C_9_H_10_O_5_	198.17		199	153			*Triticum aestivum* [87]; *Rosa acicularis* [16]; *Rosa rugosa* [31]; *Rhus coriaria* [59]; *Juglans mandshurica* [61]
80	Phenolic acid	**3-O-caffeoylshikimic acid** [3-CSA]	C_16_H_16_O_8_	336.29		337	319; 290; 191	273; 237; 199		*Inula viscosa* [88]; *Phoenix dactylifera* [34]
81	Phenolic acid	**Salvianolic acid G**	C_18_H_12_O_7_	340.28		341	323	276	164	*Salvia miltiorrhiza* [89]; Mentha [64]; *Terminalia arjuna* [90]
82	Phenolic acid	**Caffeic acid derivative**	C_16_H_18_O_9_Na	377.29	377		341; 195	179	119	Bougainvillea [91]
83	Phenolic acid	**Caffeic acid-O- hexoside derivative**	C_20_H_16_O_12_	448.33	447		301	271; 211; 179; 151	151	Lemon, Passion fruits [78]; Embelia [30]
84	Phenolic acid	**Ellagic acid-*O*-hexoside**	C_20_H_16_O_13_	464.33		465	303; 235	258	210	*Pelargonium endlicherianum* [15]; Eucalyptus [25]; *Punica granatum* [57]; *Carpinus betulus* [84]; *Terminalia arjuna* [90]
85	Stilbenes	**3-Hydroxyresveratrol** [Piceatannol]	C_14_H_12_O_4_	244.24		245	217	199		*Rosa rugosa*; *Rosa davurica* [60]; *G. linguiforme*; *F. pottsii* [62]
86	Stilbenes	**Resveratrol** [trans-Resveratrol; 3,4′,5-Trihydroxystilbene]	C_14_H_12_O_3_	228.24		229	209	139		Embelia [30]; *G. linguiforme*; *F. glaucescens*; *F. herrerae*; *F. pottsii* [62]
87	Coumarin	**Esculetin** [3,7-Dihydroxylcoumarin; Cichorigenin]	C_9_H_6_O_4_	178.14		179	151; 133; 123	123		*Artemisia annua* [92]; Basilic; Rosemary; Thymus vulgaris, Mint; Salvia [37]
88	Coumarin	**Umbelliferone hexoside**	C_15_H_16_O_8_	324.28		325	307	287		*Lonicera caerulea* [24]; *G. linguiforme*; *F. pottsii* [62];
89	Lignans	**Hinokinin**	C_20_H_18_O_6_	354.35		355	337	319	300	Lignans [93]; *Triticum aestivum* [82,87]
90	Lignans	**Pinoresinol**	C_20_H_22_O_6_	358.38		359	341; 191	277		*Triticum aestivum* L. [10,87]; *Punica granatum* [57]; *Rosa rugosa*; *Rosa davurica* [60]; Lignans [93]
91	Lignans	**Syringaresinol**	C_22_H_26_O_8_	418.44		419	383; 355; 326; 295	298		Wheat [94]; *Triticum aestivum* [82]; Lignans [93]
92	Lignans	**Pinosylvin (double glycosylation)**	C_26_H_32_O_12_	536.52		537	408	351; 149	308; 237	*Triticum aestivum* [87]; *Triticum aestivum* L. [10]
93	Dihydrochalcone	**Phloretin** [Dihydronaringenin; Phloretol]	C_15_H_14_O_5_	274.26		275	256	212	212	*Loropetalum chinense* [17]; Eucalyptus [25]; *G. linguiforme*; *F. pottsii* [62]; *Eleocharis dulcis* [66]
		**Others**								
94	Aliphatic amino acid	**L-Glutamic acid [L-Glutamate]**	C_5_H_7_NO_4_	145.11		146	127			*Medicago truncatula* [95]
95	Amino acid	**Phenylalanine** [L-Phenylalanine]	C_9_H_11_NO_2_	165.18		166	120			*Camellia kucha* [14]; *Rosa acicularis* [16]; *Juglans mandshurica* [61]; *G. linguiforme*; *F. pottsii* [62]; *Medicago truncatula* [95]
96	Amino acid	**L-theanine** [Theanine; Theanin; N-Ethyl-L-glutamine]	C_7_H_14_N_2_O_3_	174.19		175	157; 135; 117			*Rosa amblyotis* [16]; *Camellia kucha* [14]; *Glycine soja* [20]; *Ribes aureum*, *Ribes triste* [27]
97	Vitamin	**L-Ascorbic acid** [Vitamin C]	C_6_H_8_O_6_	176.12		177	145	117		Strawberry, Papaya, Lemon, Passion fruits [78]; *Phoenix dactylifera* [34]
98		**8-Hydroxy-tetradec-(9E)-ene-11,13-diyn-2-one**	C_14_H_18_O_2_	218.29	217		180	143		*Rosa acicularis* [16]
99	Carboxylic acid	**Myristoleic acid** [Cis-9-Tetradecanoic acid]	C_14_H_26_O_2_	226.35		227	209; 165	121		*Maackia amurensis* [96]; *G. linguiforme*; *F. glaucescens*; *F. pottsii* [62]
100	Sesquiterpenoid	**Atractylenolide I**	C_15_H_18_O_2_	230.30		231	203; 185; 162; 139	185; 139	139	Chinese herbal formula Jian-Pi-Yi-Shen pill [65]
101	Sulfur-contained amino acid cystine	**Cystine**	C_6_H_12_N_2_O_4S2_	240.30	239		195; 151	150	135	*Triticum aestivum* [97]
102	Trihydroxy anthraquinone	**Chrysophanol** [Chrysophanolic acid; 3-Methylchrysazin]	C_15_H_10_O_4_	254.23		255	227; 209	209		*Dryopteris fragrans* [98]; Chinese herbal formula Jian-Pi-Yi-Shen pill [65]
103		**Tanshinone I [Tanshinone A; Tanshinone]**	C_18_H_12_O_3_	276.28		277	259; 115	241; 115		*Radix Salviae* [99]
104	Omega-3 fatty acid; octadecatetraenoic acid	**Stearidonic acid** [6,9,12,15-Octadecatetraenoic acid; Moroctic acid]	C_18_H_28_O_2_	276.41		277	260	215; 115	128	*Salviae Miltiorrhizae* [100]; *Rhus coriaria* [59]; *G. linguiforme*; *F. pottsii* [62]; Jatropha [63]
105	Omega-3 fatty acid	**Linolenic acid** (Alpha-Linolenic acid; Linolenate)	C_18_H_30_O_2_	278.42		279	261	115		*Salviae Miltiorrhizae* [100]; *Glycine soja* [20]; Jatropha [63]
106		**Linoleic acid amide**	C_18_H_33_NO	279.46		280	262	244; 118	196	Propolis [33]; *Rhus coriaria* [59]
107	Naphthoquinone	**Rhein** [Monorhein; Rheic acid; Rhubarb Yellow]	C_15_H_8_O_6_	284.22		285	257; 149	117		*Juglans mandshurica* [61]; Chinese herbal formula Jian-Pi-Yi-Shen pill [65]
108	Triterpenoid	**3,4-Dihydroxyestran-17-one**	C_18_H_28_O_3_	292.41		293	275; 177	257	213	*Juglans mandshurica* [61]
109	Fatty acid	**Methyl linolenate**	C_19_H_32_O_2_	292.45		293	261	243; 187; 133	187; 159	*Apium graveolens* [101]
110	Triterpenoid	**Abietan-18-ol**	C_20_H_36_O	292.49		293	275; 177	257	213	*Juglans mandshurica* [61]
111	Diterpenoid naphthoquinone	**Tanshinone IIA** [Tanshinone II; Tanshinone B]	C_19_H_18_O_3_	294.34		295	277	259; 161	194	*Radix Salviae* [99]; Chinese herbal formula Jian-Pi-Yi-Shen pill [65]
112	Fatty acid	**Methyl linoleate**	C_19_H_34_O_2_	294.47		295	244; 159	149		*Apium graveolens* [101]
113	Tyramines	***N-trans*** **-feruloyl tiramine**	C_18_H_19_NO_4_	313.34		314	268; 240; 194; 151	240; 167; 125	194	Propolis [33]; *G. linguiforme*; *A. cordifolia*; *F. pottsii* [62]; Jatropha [63]; Bougainvillea [91]
114	Cyclohexenecarboxylic acid	**4-O-*p*-coumaroyl shiikimic acid**	C_16_H_16_O_7_	320.29		321	275	257; 197	238	*Phoenix dactylifera* [34]
115	Oxylipins	**13- Trihydroxy-Octadecenoic acid** [THODE]	C_18_H_34_O_5_	330.45	329		229; 171	209; 155	183	*Bituminaria* [102]; *Phoenix dactylifera* [34]; Jatropha [63]
116	Alpha,omega-dicarboxylic acid	**Eicosatetraenedioic acid**	C_20_H_30_O_4_	334.44		335	289	273; 288		*Lonicera caerulea* [24]; *G. linguiforme*; *F. pottsii* [62]
117	Cyclohexenecarboxylic acid	**Caffeoyl shikimic acid**	C_16_H_16_O_8_	336.29		337	291	273	199	*Carpinus betulus* [84]
118		**Docosenoic acid** [2-Docosenoic acid]	C_22_H_42_O_2_	338.56		339	321; 209	245	121	*A. cordifolia*; *F. glaucescens*; *F. herrerae G. linguiforme* [62]
119	Diarylheptanoid	**4,16-Dihydroxy-17-methoxy-2-oxatricyclo [13.2.2.1-3,7-]icosa-1(17),3(20),4,6,15,18-hexaen-10-one**	C_20_H_22_O_5_	342.38		343	325; 297	297		*Juglans mandshurica* [61]
120	Naphthoquinone	**8,8′-Dihydroxy-2,2′-binaphthalene-1,1′,4,4′-tetrone**	C_20_H_10_O_6_	346.28		347	319; 158	291	261	*Juglans mandshurica* [61]
121		**1,8,11-Trihydroxy-3-methoxy-10-methyl-5,12-tetracenedione**	C_20_H_14_O_6_	350.32		351	333	314; 257	271	*Juglans mandshurica* [61]
122		**Trihydroxy eicosatetraenoic acid**	C_20_H_32_O_5_	352.46		353	177; 145	145		*F. glaucescens*; *G. linguiforme*; *F. pottsii* [62]
123	Naphthoquinone	**3,3′,5,5′-Tetrahydroxy-2,2′-binaphthalene-1,1′,4,4′-tetrone**	C_20_H_10_O_8_	378.28		379	361	344	302; 164	*Juglans mandshurica* [61]
124	Unsaturated fatty acid	**Pentacosenoic acid**	C_25_H_48_O_2_	380.64		381	362; 253; 191	270; 226		*F. glaucescens*; *G. linguiforme*; *F. pottsii* [62]
125	Sterol	**Campesterol** [Dihydrobrassicasterol]	C_28_H_48_O	400.68						*Cicer arietinum* [103]; *A. cordifolia*; *C. edulis*; *G. linguiforme*; *F. pottsii* [62]
126	Triterpenoid	**Squalene** (Trans-Squalene; Spinacene; Supraene)	C_30_H_50_	410.71		411	265; 177	248; 177		Squalene [104]
127	Anabolic steroid; Androgen; Androgen ester	**Vebonol**	C_30_H_44_O_3_	452.66		453	435; 336; 209	336; 226	209	*Hylosereus polyrhizus* [105]; *Rhus coriaria* [59]
128	Triterpenoid	**1-Hydroxy-3-oxours-12-en-28-oic acid**	C_30_H_46_O_4_	470.68		471	453; 359; 209	436; 388; 343; 265; 208		Pear [106]; *Rosa amblyotis* [16]
